# Influence of climate on the number of hospitalizations for nephrolithiasis in urban regions in Brazil

**DOI:** 10.1590/2175-8239-JBN-2019-0155

**Published:** 2020-05-08

**Authors:** João de Abreu, Sebastião Rodrigues Ferreira

**Affiliations:** 1 Universidade de Uberlândia, Departamento de Medicina Interna, Uberlândia, Minas Gerais, Brasil

**Keywords:** Nephrolithiasis, Kidney Calculi, Lithiasis, Climate Change, Climate, Nefrolitíase, Cálculos Renais, Litíase, Mudança Climática, Clima

## Abstract

**Introduction:**

Nephrolithiasis has a worldwide prevalence of approximately 5 to 15%, and its occurrence is associated with age, sex, race, dietary habits, geographic location, climatic conditions, and other factors. The objective of the present study was to determine the association between climate and the number of hospitalizations for nephrolithiasis (NH) in Brazilian cities located in different climatic regions.

**Methods:**

We analyzed data from cities with tropical and subtropical climates. The effects of the lowest (LT), mean (MT), and highest (HT) monthly temperatures and relative humidity of the air (RH) were assessed.

**Results:**

A positive association was found between the number of hospitalizations for nephrolithiasis and temperature ((LT x NH; R^2^=0.218; P<0.0001) (MT x NH; R^2^=0.284; P<0.0001) (HT x NH; R^2^=0.317; P<0.0001)), and a negative association was found between the number of hospitalizations for nephrolithiasis and the relative humidity (RH x NH; R^2^=0.234; P<0.0001). Interactions were also observed between MT and RH with respect to their effects on the NH, as described by a linear model (NH = 4.688 + 0.296 x MT - 0.088 x RH). The NH was higher in cities with tropical climates than in cities with subtropical climates (82.4 ± 10.0 vs 28.2 ± 1.6; P<0.00001).

**Conclusion:**

There is an association between the NH and variations in temperature and relative humidity.

## Resumo

**Introdução:**

A prevalência mundial da nefrolitíase situa-se entre 5% e 15%. Sua ocorrência está associada a idade, sexo, raça, hábitos alimentares, localização geográfica, condições climáticas e outros fatores. O objetivo do presente estudo foi determinar a associação entre clima e número de internações por nefrolitíase (IN) em cidades brasileiras localizadas em diferentes regiões climáticas.

**Métodos:**

Analisamos dados de cidades com climas tropicais e subtropicais. Nossa avaliação considerou os efeitos das temperaturas mensais mais baixas (TMB), sua média (TM) e mais altas (TMA) e da umidade relativa do ar (UR).

**Resultados:**

Foi identificada associação positiva entre o número de internações por nefrolitíase e temperatura ((TMB vs. IN; R^2^ = 0,218; P<0,0001) (TM vs. IN; R^2^ = 0,284; P<0,0001) (TMA vs. IN; R^2^ = 0,317; P<0,0001)) e associação negativa entre o número de internações por nefrolitíase e umidade relativa do ar (UR vs. IN; R^2^ = 0,234; P <0,0001). Também foram observadas interações entre TM e UR com relação aos seus efeitos sobre a IN, conforme descrito por um modelo linear (IN = 4,668 + 0,296 x TM - 0,088 x UR). IN foi mais acentuada nas cidades com climas tropicais do que nas cidades com climas subtropicais (82,4 ± 10,0 vs. 28,2 ± 1,6; P<0,00001).

**Conclusão:**

Existe associação entre IN e variações de temperatura e umidade relativa.

## Introduction

Nephrolithiasis (NL) is a highly prevalent disease worldwide, with rates ranging from 7.1% for women to 10.6% for men in North America^1^. Epidemiological data from Spain^2^, Germany^3^, Japan^4^, and Italy^5^ revealed incidence rates for kidney stones of 114-720 per 100,000 individuals and prevalence rates of 1.7-14.8%, and in nearly all countries, the rates seem to be rising^6^^,^ 7. In Japan, the prevalence has steadily increased^4^, which is similar to trends observed in many other countries indicating a low incidence in childhood and the elderly and peak incidence in the fourth to sixth decades of life^8^.

In Brazil, the prevalence can be estimated from data from the Public Health System of Brazil, which is named the Sistema Único de Saúde (SUS)^9^ and supposedly provides health coverage for all citizens of the country^10^. The SUS has a longitudinal hospital inpatient database (Sistema de Informação Hospitalar-SIH/SUS), which contains records from discharges for all cities and regions of the country^11^. Unfortunately, current scientific articles regarding the frequency of kidney stone episodes in Brazil are limited^12^, and studies describing changes in climate and hospitalizations are lacking.

Overall, there has been a persistent male predominance in the prevalence and incidence of kidney stones^13^, but the male/female ratio has been reduced over the last few decades^14^. Race^15^, dietary habits^16^^,^ 17, genetics^18^, occupational factors^19^, geographic location^20^, and climatic conditions^21^ are considered risk factors for NL. In addition to medical problems, NL is usually associated with high rates of work absenteeism, an increased number of days of home rest, and prolonged hospital stays. The costs of medical and surgical procedures plus the costs from the loss of work productivity are considerable^12^^,^ 22.

Some studies report that NL is more frequent in places with higher temperatures when associated with reduced water intake, resulting in more concentrated urine and facilitating the nucleation of renal calculi^20^^,^ 23. In addition, the season of the year has been linked to the incidence and prevalence of NL, with the hottest months of the year being notable for the increased development of kidney stones^24^^,^ 25. Several reports describe an association between the season and the level of urine oxalate^26^, with urine oxalate and calcium^27^ excretion being significantly increased in the summer compared with winter. Conversely, other studies have failed to show such associations^28^. On the other hand, studies of the demographic and geographic variability of the incidence of kidney stones reveale higher rates of hospitalization in regions with a warmer climate and lower rates in colder regions^23^^,^ 29.

Brazil, a country of continental proportions, has broad variation in temperature between the north (tropical area) and the south (subtropical area) regions of the country. The objective of the present study was to assess the association between climate and the number of hospitalizations due to NL (NH) in Brazilian cities located in different climatic conditions.

## Methods

This retrospective cohort study analyzed NH over the period from January 1^st^ 2010 to December 31^st^ 2015. Using the public domain database of the Brazilian Ministry of Health, which has been made available by the Department of Informatics of the SUS (DATASUS) on the website of the Decentralized Hospital Information System (SIHD), we selected the necessary software and files^30^. We included cities with a population of more than 300,000 inhabitants (inhab), according to data from the Brazilian Institute of Geography and Statistics (IBGE) database^31^, located in tropical and subtropical climate regions with permanent records in the SIHD2^30^.

**Figure 1:**
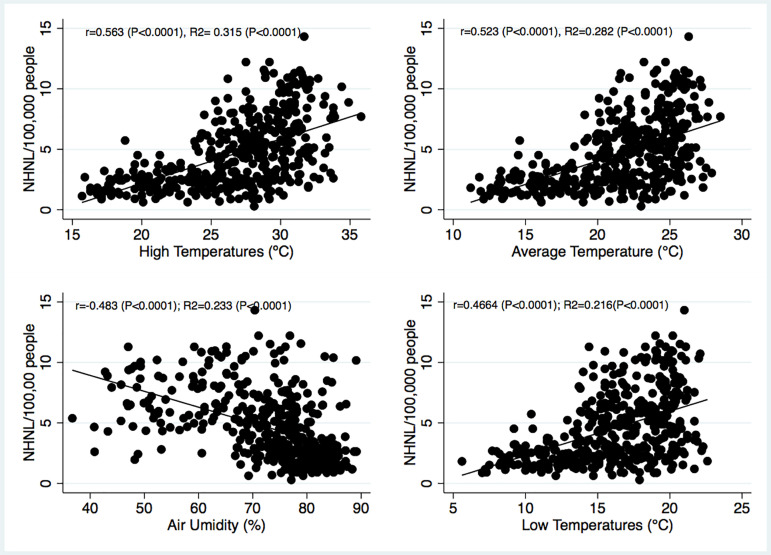
Associations of NH with the lowest (LT), mean (MT), and highest (HT) temperatures and air humidity (RH).

The selected cities in tropical regions were Uberlândia/Minas Gerais (MG) (Latitude (LA): -18.9113, Longitude (LO): -48.2622 18 ° 54′ 41″ South (S), 48° 15′ 44″ West (W)), Ribeirão Preto/São Paulo (SP) (La: -21.1767, Lo: -47.8208 21 ° 10′ 36″ S, 47 ° 49′ 15″ W) and São José do Rio Preto/SP (La: -20.8202, Lo: -49.3797 20 ° 49′ 13″ S, 49 ° 22′ 47″ W). For subtropical climates, we selected the cities of Porto Alegre/Rio Grande do Sul (RS) (La: -30.0277, Lo: -51.2287 30 ° 1′ 40″ S, 51 ° 13′ 43″ W), Caxias do Sul/RS (La: -29.1678, Lo: -51.1794 29 ° 10′ 4″ S, 51 ° 10′ 46″ W) and Pelotas/RS (La: -31,776, Lo: -52.3594 31 ° 46′ 34″ S, 52 ° 21′ 34″ W). The lowest (LT), mean (MT), and highest (HT) temperatures and relative humidity (RH) of each city were recorded monthly and reported in degrees Celsius and as a percentage, respectively. The MT was computed as the arithmetic average of LT and HT. The temperature and RH data were obtained from the National Institute of Meteorology (INMET) 32. The correlations between NH and the temperatures are shown in Figure 1, where the total number of months computed was indicated by the product of six cities, 12 months for each city, and six years (6 cities X 12 months X 6 years = 432 months).

NH was calculated as 100,000 inhab/month by using records with the following International Classification of Diseases 10 codes, version 2016^33^: N20 - calculus of kidney and ureter; N21 - calculus of lower urinary tract; N22 - calculus of urinary tract in diseases classified elsewhere; and N23 - unspecified renal colic. We analyzed NH by sex and among nine age groups that ranged from children under 1 year of age to adults over 65 years of age.

### Statistical Analysis

We assessed the normality of the data set using the Kolmogorov-Smirnov test. All variables were normally distributed, so we report the data as the means and standard deviations. Two groups were compared with Student’s t-test, whereas three or more groups were compared with analysis of variance (ANOVA) and the Bonferroni post-test. Pearson’s coefficient was calculated for the relationship between two continuous or ordinal variables. Univariate and multivariate linear regressions were used to quantify the association between NH and climatic data. To determine the interactions between MT, RH, and NH, we used a multiple linear regression model, in which the model variables were previously tested and gradually added into the global analysis (Stepwise), with NH as the dependent variable and MT and RH as independent variables. A p value < 0.05 (95% confidence interval (CI)) were considered significant for all calculations. The software used was STATA version 15 (StataCorp LP, College Station, Texas).

## Results

In total, there were 8,119 admissions in tropical and 4,388 admissions in subtropical cities (Table 1). The distribution of NH was 3,921 (48.2%) for males and 4,198 (51.8%) for females in tropical cities (P = 0.045), while in the subtropical region, it was 2,268 (52.1%) for males and 2,120 (47.9%) for females (P = 0.058) with male/female ratios of 0.9 and 1.1, respectively. It was noted that 727 readmissions, corresponding to 27% of hospitalizations, occurred only in Uberlândia city, while in other cities, it was not possible to calculate the number of readmissions for NL in the period studied.

**Table 1:** Number of hospitalizations in the tropical and subtropical cities for gender, race, and age ranges.

	Tropical cities	Subtropical cities	*P value*
Total Population	1,692,660	2,233,680	
Total NH	249.4 ± 27.54	84.3 ± 4.36	<0.0001
Gender			
Male	119.7 ± 17.03	44.3 ± 2.57	<0.0001
Female	131.3 ± 10.91	40.4 ± 2.13	<0.0001
Race			
White	182.2 ± 15.90	70.0 ± 2.97	<0.0001
B/B/I	35.2 ± 8.20	7.5 ± 0.97	<0.0001
Asian	1.8 ± 0.58	0.4 ± 0.33	<0.0001
W/N	30.4 ± 9.35	6.0 ± 1.69	<0.0001
Age Range (years)			
<1	0.12 ± 0.21	0.0 ± 0.03	0.037
1 to 4	0.53 ± 0.50	0.1 ± 0.11	0.005
5 to 14	3.1 ± 1.33	1.2 ± 0.50	<0.0001
15 to 24	23.9 ± 3.12	7.1 ± 0.91	<0.0001
25 to 34	48.8 ± 5.17	17.6 ± 2.74	<0.0001
35 to 44	54.3 ± 8.79	18.9 ± 1.84	<0.0001
45 to 54	57.0 ± 7.23	19.4 ± 2.53	<0.0001
55 to 64	33.1 ± 2.49	11.2 ± 1.01	<0.0001
≥65	27.2 ± 4.06	8.6 ± 0.95	<0.0001

NH: Number of hospitalizations due to renal stones/100,000/ month; B/B/I: Black, Brown, Indigenous; W/N: Without notification.

**Figure 2:**
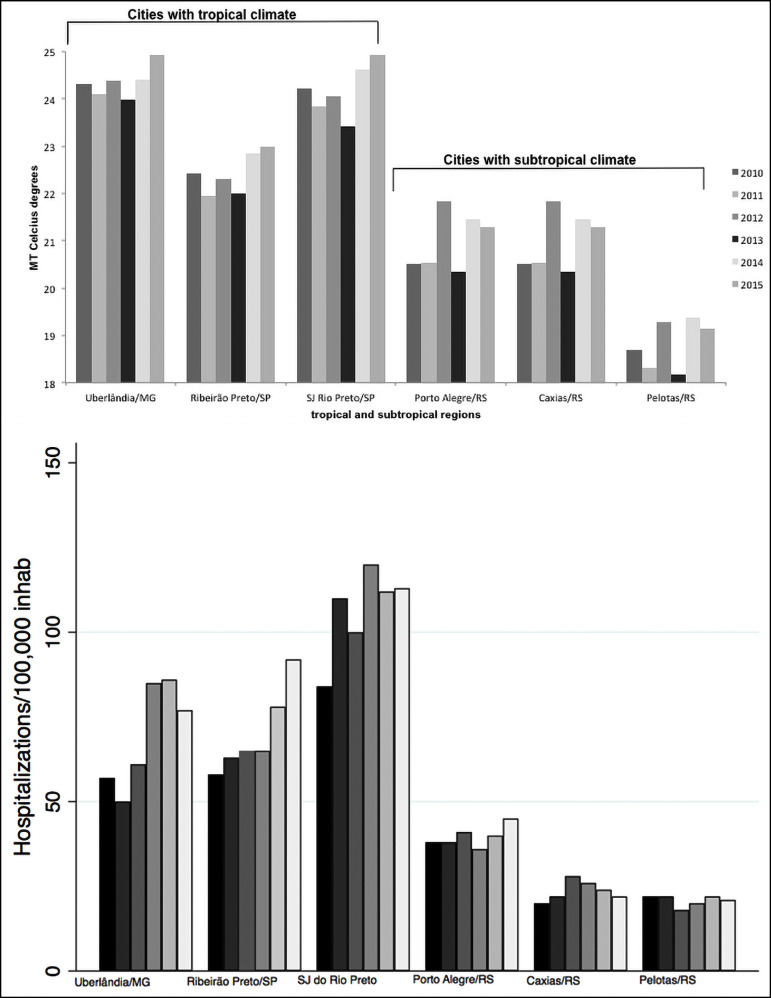
Association of the mean temperature and NH from 2010 to 2015 in tropical and subtropical regions (SP: Sao Paulo state; RS: Rio Grande do Sul state)

Figure 1 shows the significant positive coefficients for the associations between NH and LT, MT, and HT. A significant negative coefficient were observed for the association between NH and RH. Figure 2 shows the annual cumulative NH and the mean annual temperatures from 2010 to 2015 in the tropical and subtropical cities.

The interactions between MT, RH, and NH were assessed using multivariate regression, with NH as the dependent variable and MT and RH as the independent variables, as shown in equation 1:

NH=4.688+0.296xMT–0.088xRH,

*where:*

NH is a number of hospitalizations for nephrolithiasis per 100,000 inhabitants/month;

MT is an arithmetic mean of LT and HT for each city (monthly) and

RH is a monthly relative humidity in each city.

**Figure 3:**
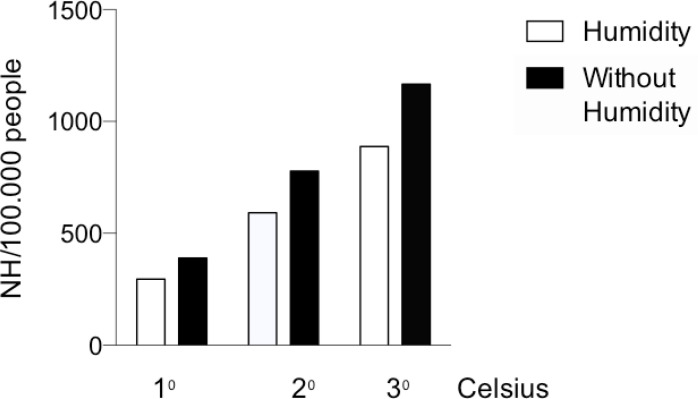
Expected increases in NH due to increases in global MT, with and without controlling for RH, for a population of 100 million inhabitants

Based on the above equation, an elevation of 1ºC in the MT in the presence of constant RH resulted in 296 new hospitalizations per 100 million people, and if we disregard the RH in the calculations, the result would be 389 new hospitalizations per 100 million people (Figure 3).

## Discussion

Our study showed significant positive associations between NH and LT as well as MT and HT and a significant negative association between NH and RH, showing that climatic changes can alter the number of new hospitalizations and/or readmissions due to NL. The influence of climate on the prevalence of NL was observed in studies by Eisner et al.^21^ and Ross et al.^29^, who demonstrated a higher incidence of renal calculi in the summer than in winter. These data are similar to those found by our study, which demonstrated a higher NH in cities with tropical climates compared to that in cities with subtropical climates. The possible causes suggested by the authors for the increased incidence of NL in patients in hot climates are increased urinary calcium levels, supersaturation of calcium oxalate and calcium phosphate, and decreased urinary sodium excretion^21^, which are independent of the RH and season. In Brazil, temperature variations are not as significant across seasons, but there is evident variation between tropical and subtropical regions (Figure 2). We carefully designed our multivariate analysis to include covariates such as temperature and air humidity to determine the relationships between these variables and NH. We found that higher temperature was significantly associated with increased NH. Furthermore, increased air humidity was also associated with decreased NH. According to Equation 1, an increase in MT in the presence of low RH would be the worst setting for people susceptible to forming kidney stones. If we extrapolate these results to the Brazilian population, which according to data from the IBGE is comprised of approximately 200 million people^31^, there will be approximately 592 new hospitalizations per month or 7,104 new NL hospitalizations per year due to an increase of 1ºC in the monthly MT (Figure 3). Long-term occurrence of high-temperature summers should be the focus for the development of government campaigns to educate the population on the possibility of kidney stones and suggest increased water intake and working in humidified environments. Such measures could be justified on the grounds that the addition of new hospitalizations for renal lithiasis would entail additional costs that would perhaps not be foreseen in annual budgets.

Ross et al.^29^ demonstrated that the risk of renal calculus formation is better predicted by a combination of temperature and RH than when these two variables are analyzed separately. Our findings agree with those of the Ross study, as we found that temperature and RH may be simultaneously associated with NH, which was demonstrated by multivariate regression (Equation 1) in which MT and RH were independent predictors of NH (Figure 3). The Intergovernmental Panel on Climate Change (IPCC) in 2007 predicted an increase in the planetary annual mean temperature, which will result in an increased number of hot days and cold nights on almost the entire planet surface by the end of the 21st century^34^.

The influence of probable global warming on the prevalence of NL was also investigated by Brikowski et al.^23^, who reported that in the event of a sudden increase in temperature, the risk of NL would increase. These authors used linear and nonlinear models to predict NL risk in regions of the United States. In contrast, our study used only multivariate linear models. This could generate some discrepancies between the results.

Conflicting data have been reported in several studies about the gender distribution of NL. In Italy, the male/female ratio was 1.2^5^, and in the US, the ratio ranged from 0.5 to 1.8 in a single region^35^. In our study, we observed a male/female ratio of 0.9 in cities with a tropical climate and 1.1 in those with a subtropical climate. The reason for the surge in kidney stone disease in women is not precisely understood, but some have speculated that it might be attributable to changes in lifestyle and diet, resulting in an increased risk of obesity among women, which is a known risk factor for kidney stone formation^14^. Among adults, the occurrence of nephrolithiasis by age group follows a normal distribution, and the first symptomatic episode typically occurs at 35 years of age for men and 30 years for women^8^. This age pattern is similar to that found in the present study.

Our study has some limitations, including the fact that it was not possible to determine the number of readmissions for NL in all cities surveyed. Readmissions were analyzed in only one city with a tropical climate (Uberlândia/MG), and the values found there may be different from those in other cities. Another limitation was the use of linear statistical models in our study, since nonlinear models may be more appropriate. This study is retrospective and is therefore subject to the shortcomings of a non-prospective study design. We used average temperature and mean humidity for our analysis; however, we were unaware of the variations between individuals in terms of time spent outdoors and exposure to these variations on a day-to-day basis. In addition, we did not analyze dietary intake, and this could theoretically affect NH associated with climate modifications. In conclusion, NH in Brazil seems to depend on climatic variations according to the region in which the patient lives.
